# Functional Identification of a Novel Gene, *moaE*, for 3-Succinoylpyridine Degradation in *Pseudomonas putida* S16

**DOI:** 10.1038/srep13464

**Published:** 2015-08-25

**Authors:** Yi Jiang, Hongzhi Tang, Geng Wu, Ping Xu

**Affiliations:** 1 State Key Laboratory of Microbial Metabolism, and School of Life Sciences & Biotechnology, Shanghai Jiao Tong University, Shanghai 200240, People’s Republic of China; 2 Joint International Research Laboratory of Metabolic & Developmental Sciences, Shanghai Jiao Tong University, Shanghai 200240, People’s Republic of China

## Abstract

Microbial degradation of *N*-heterocyclic compounds, including xanthine, quinoline, nicotinate, and nicotine, frequently requires molybdenum hydroxylases. The intramolecular electron transfer chain of molybdenum hydroxylases consists of a molybdenum cofactor, two distinct [2Fe-2S] clusters, and flavin adenine dinucleotide. 3-Succinoylpyridine monooxygenase (Spm), responsible for the transformation from 3-succinoylpyridine to 6-hydroxy-3-succinoylpyridine, is a crucial enzyme in the pyrrolidine pathway of nicotine degradation in *Pseudomonas*. Our previous work revealed that the heterotrimeric enzyme (SpmA, SpmB, and SpmC) requires molybdopterin cytosine dinucleotide as a cofactor for their activities. In this study, we knocked out four genes, including PPS_1556, PPS_2936, PPS_4063, and PPS_4397, and found that a novel gene, PPS_4397 encoding *moaE*, is necessary for molybdopterin cytosine dinucleotide biosynthesis. Resting cell reactions of the *moaE* deletion mutant incubated with 3 g l^−1^ nicotine at 30 °C resulted in accumulation of 3-succinoylpyridine, and the strain complemented by the *moaE* gene regained the ability to convert 3-succinoylpyridine. In addition, reverse transcription-quantitative polymerase chain reaction analysis indicated that the transcriptional levels of the genes of *moaE, spmA,* and *spmC* of *Pseudomonas putida* S16 were distinctly higher when grown in nicotine medium than in glycerol medium.

Large quantities of tobacco wastes containing high concentration of nicotine are produced during tobacco manufacturing yearly^1^. Nicotine is well known to be harmful to human health and can cross biological membranes and blood-brain barrier easily^2^. Microorganisms such as *Pseudomonas* are important for degrading nicotine, and the pyrrolidine pathway of nicotine degradation has been systematically unraveled in *Pseudomonas putida* strain S16^3^,^4^,^5^,^6^. The pyrrolidine ring is first dehydrogenated to *N*-methylmyosmine, followed by spontaneous hydrolysis and oxidation to form 3-succinoylpyridine (SP). The molybdenum-dependent enzyme 3-succinoylpyridine monooxygenase (Spm) catalyzes SP to 6-hydroxy-3-succinoylpyridine (HSP) (Fig. 1). This enzyme is composed of three subunits: a large subunit carrying molybdopterin cytosine dinucleotide (Mo-MCD), a middle subunit binding a flavin adenine dinucleotide (FAD) molecule, and a small subunit with two [2Fe-2S] clusters^3^. When the *spmABC* genes are expressed in *Escherichia coli*, an inactive Spm is formed that lacks the Mo-MCD cofactor. Instead, the cofactor in *E. coli* is molybdopterin guanine dinucleotide (MGD), which cannot be integrated into the functional apoprotein Spm^3^.

High-valent molybdenum has been identified as the mononuclear active site of a series of oxidoreductases, on account of its water solubility and general catalytic activity to transfer an oxygen atom either to or from the electron acceptor or donor^7^,^8^,^9^. SpmABC in the pyrrolidine pathway is attributed to typical prokaryotic molybdenum-containing hydroxylases that are found in the biodegradation pathways of some *N*-heterocyclic compounds^10^. In *Arthrobacter nicotinovorans*, nicotine dehydrogenase and ketone dehydrogenase catalyze the formation of pyridine ring-hydroxylated products, and Mo-MCD is required for holoenzyme assembly of these two enzymes^11^,^12^. Significantly, the biodegradation of isonicotinate by *Mycobacterium* sp. INA1^13^ and the metabolism of nicotinate by *Bacillus niacini*^14^ also have similar hydroxylation steps to generate 2,6-dihydroxy-substituted products, which illustrates the important role of molybdenum cofactor in the metabolism of *N*-heterocyclic compounds. Therefore, it is crucial to isolate and identify the essential genes of molybdenum cofactor biosynthesis involved in the nicotine degradation of *Pseudomonas*.

In this study, we disrupted several genes that were selected by mining of the genome sequence of strain *P. putida* S16 to identify Mo-MCD synthesis-related genes. We found that PPS_4397, encoding *moaE*, was necessary for Mo-MCD biosynthesis in strain *P. putida* S16. The *moaE* deletion mutant strain *P. putida* S16d*moaE* could not grow with nicotine as the sole carbon and nitrogen source. Resting cells of *P. putida* S16d*moaE* incompletely metabolized nicotine and accumulated SP-like *P. putida* S16d*spm*^3^. Here, we identify and characterize the *moaE* gene required for the synthesis of Mo-MCD in nicotine degradation. The objective of this work was to demonstrate the unknown catabolic genes involved in the nicotine degradation pathway from *Pseudomonas*.

## Results

### Identification of genes for Mo-MCD synthesis

**Figure 2:**
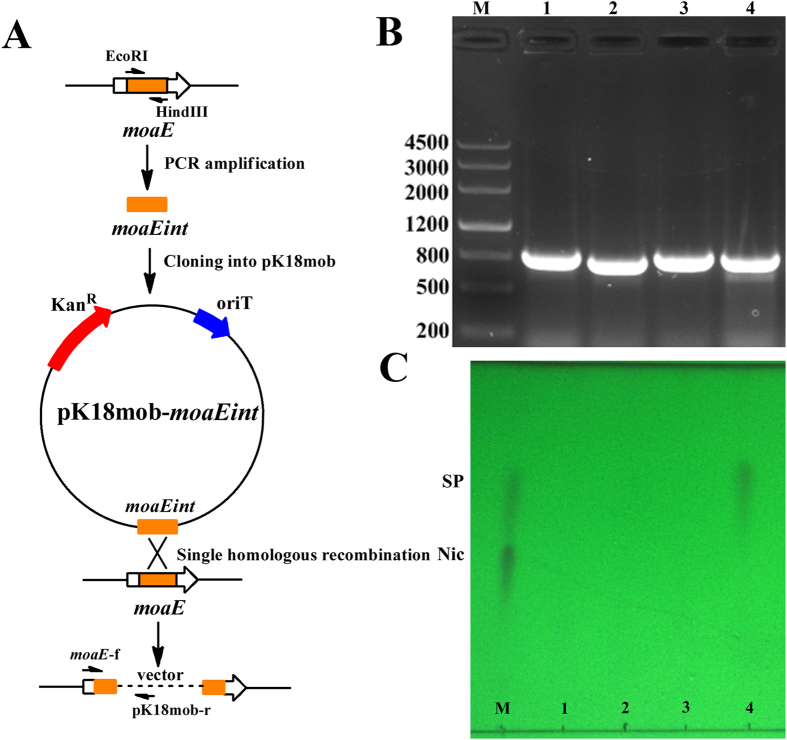
Plasmid construction and gene disrupted mutants’ confirmation by PCR. **(A)** The procedure of plasmid construction. The *moaE* gene was deleted by single homologous recombination using suicide plasmid pK18mob. **(B)** PCR analysis of gene disrupted mutants. The former primer is the corresponding upstream gene and the reverse primer is a section sequence of pK18mob. Lane M, marker; lane 1, S16dPPS_1556; lane 2, S16dPPS_2936; lane 3, S16dPPS_4063; lane 4, S16d*moaE*; **(C)** TLC analysis of the supernatant by incubation of 3 g l^−1^ nicotine and resting cells of gene disrupted mutants at 8h. Resting cells reaction condition is 30 °C and 200 rpm. Lane M, marker containing 1 g l^−1^ nicotine and 4 g l^−1^ SP as standards; lane 1, S16dPPS_1556; lane 2, S16dPPS_2936; lane 3, S16dPPS_4063; lane 4, S16d*moaE*.

Gene annotation and protein sequence alignment of strain S16 revealed several genes predicted to be potentially responsible for Mo-MCD synthesis. In consideration of the conserved biosynthetic pathway of molybdenum cofactor in all bacteria, gene annotation was quite credible. These predicted genes involved in molybdate uptake, molybdopterin biosynthesis and molybdenum cofactor further modification. Four of these genes (PPS_1556, encoding for molybdenum cofactor biosynthesis protein A; PPS_2936, encoding for molybdopterin-guanine dinucleotide biosynthesis protein A; PPS_4063, encoding for molybdenum cofactor biosynthesis protein; PPS_4397, encoding for molybdopterin biosynthesis MoaE protein) were successfully knocked out by using the suicide plasmid pK18mob, which were confirmed by PCR analysis (Fig. 2B). All the mutants were cultured in Luria-Bertani medium containing 1 g l^−1^ of nicotine and prepared as resting cells to degrade nicotine. After 8 h, the sample was immediately detected by thin-layer chromatography, and only the PPS_4397 gene (*moaE*) deletion strain could accumulate SP (Fig. 2C).

### Primary sequence alignment of *moaE*

**Figure 3:**
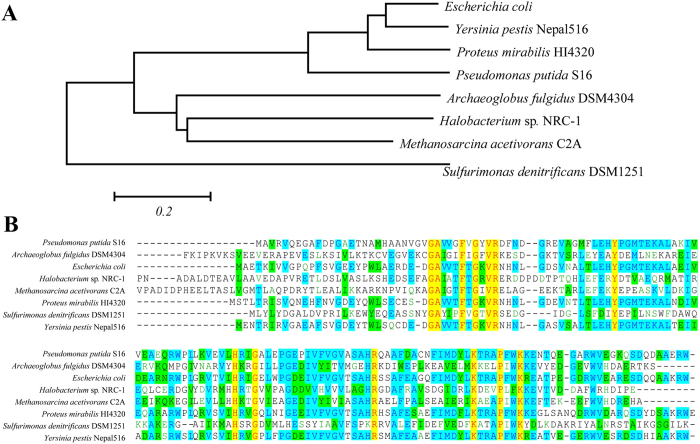
Phylogenetic tree and sequence conservation of MoaE. (**A**) Phylogenetic tree of MoaE from eight different strains constructed with the neighbor joining method using MEGA 4.1. (**B**) Multiply sequence alignment of MoaE from the different eight species using Vector NTI. Identical conservative sites are highlighted in yellow, comparatively conservative sites are highlighted in blue and green.

The amino acid sequence translated from the *moaE* gene was compared to known molybdopterin synthase large subunit (MoaE) sequences from seven different species in the National Center of Biotechnology Information data library. Phylogenetic tree construction for the individual proteins was performed using MEGA 4.1 with the neighbor joining method^15^. The results showed that MoaE from *P. putida* S16 is closely related to the ortholog from *P. mirabilis* HI4320 (Fig. 3A). Multiple sequence alignments by Vector NTI revealed that the gene product also contains reported highly conserved sites, including Arg 37, Lys 117, and Lys 124^16^ (Fig. 3B).

### Cell growth and resting cell reactions of mutant *moaE*

**Figure 4:**
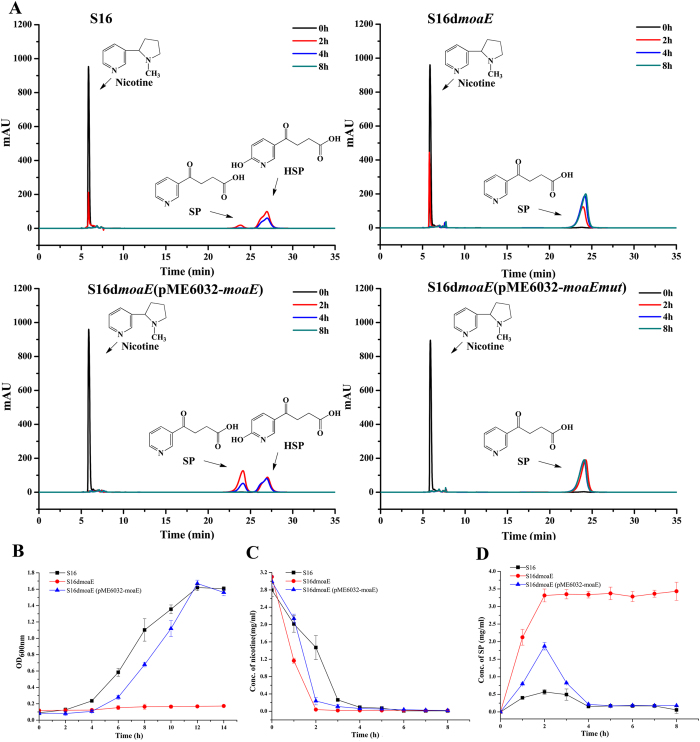
Deletion and complement of *moaE* gene. (**A**) HPLC spectrogram of intermediates produced by reaction of resting cells of *P. putida* S16, *P. putida* S16d*moaE, P. putida* S16d*moaE* (pME6032-*moaE*) and *P. putida* S16d*moaE* (pME6032-*moaEmut*) at 30 °C using 3 g l^−1^ nicotine as the substrate. black: 0 h, red: 2 h, blue: 4 h, green: 8 h. (**B**) Growth curve of *P. putida* S16 (■), the *moaE* gene deletion mutant *P. putida* S16d*moaE* (•) and *P. putida* S16d*moaE* (pME6032-*moaE*) (▲) with nicotine as the sole carbon and nitrogen source at 30 °C and 200 strokes (rpm). **(C)** HPLC analysis of nicotine degradation by resting cells of *P. putida* S16 (■), *P. putida* S16d*moaE* (•)*, P. putida* S16d*moaE* (pME6032-*moaE*) (▲) at 30 °C. **(D)** HPLC analysis of SP formation by resting cells of *P. putida* S16 (■), *P. putida* S16d*moaE* (•)*, P. putida* S16d*moaE* (pME6032-*moaE*) (▲) at 30 °C.

Cell growth and resting cell reaction evaluations of the *moaE* gene deletion mutant were performed and monitored by high-performance liquid chromatography (Fig. 4A). The mutant could not grow in the nicotine medium, whereas the wild-type strain S16 grew rapidly in this medium (Fig. 4B). In addition, resting cells of the *moaE* deletion mutant transformed 3 g l^−1^ nicotine into SP over 2 h, and no subsequent products were detected (Fig. 4C). In order to further verify gene function, the shuttle plasmid pME6032-*moaE* was transferred into *P. putida* S16*dmoaE*. The transformant, *P. putida* S16d*moaE* (pME6032-*moaE*), recovered the ability to grow with nicotine as the sole carbon and nitrogen source, although the growth rate was slightly slower than that of the wild-type strain (Fig. 4B). Resting cells of strain *P. putida* S16d*moaE* (pME6032-*moaE*) could also convert SP to downstream metabolites and accumulated only a small amount of SP at 2 h (Fig. 4D). However, resting cells of *P. putida* S16d*moaE* (pME6032-*moaEmut*) continued to accumulate SP at 8 h (Fig. 4A).

### RT-qPCR analysis of *moaE, spmA,* and *spmC*

**Figure 5:**
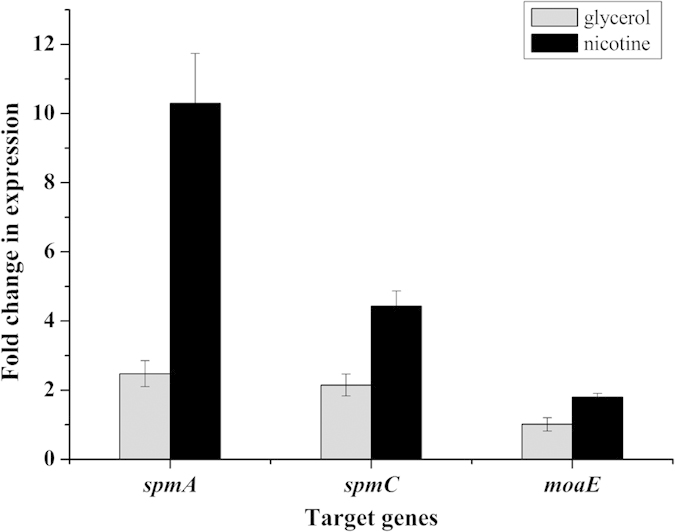
Confirmation of transcriptional levels of differentially expressed proteins that were related to SP degradation. RT-qPCR analysis of target gene transcripts produced in *P. putida* S16 grown in the nicotine (black) or glycerol (grey) medium. mRNA expression levels of *spmA, spmC* and *moaE* were measured using RT-qPCR and the 2^ΔΔCT^ method, while the 16S rRNA gene was used as the reference gene. Results presented in the bar chart are the means of three parallel experiments, and error bars illustrate the standard deviations.

RT-qPCR results showed that all target genes related to Spm holoenzyme activity were distinctly up-regulated from *P. putida* S16 grown in nicotine medium relative to those grown in the glycerol medium. Only a minimal difference was found for expression levels of *moaE,* which was upregulated 1.8-fold, whereas the mRNAs of *spmA* and *spmC* were 4.1- and 2.1-fold upregulated, respectively, with nicotine induction (Fig. 5). These results demonstrated that the transcriptional levels of *moaE, spmA,* and *spmC* of *P. putida* S16 in nicotine medium were distinctly higher than those in the glycerol medium, indicating that these genes are nicotine-induced.

## Discussion

Nicotine, the most harmful organic ingredient from tobacco, is a serious threat to human health along with the prosperity of the tobacco industry^17^. Since *P. putida* can efficiently degrade nicotine as a sole carbon and nitrogen source^18^,^19^,^20^, analysis of the crucial enzymes in the nicotine metabolic pathway will offer useful guidance for the biological treatment of tobacco wastes and accumulation of valuable intermediate products. Nicotine is classified into *N*-heterocyclic compounds, which generally require dehydrogenases to catalyze hydroxylation of the aromatic ring^21^. These dehydrogenases are frequently composed of multiple subunits, one of which binds the molybdopterin dinucleotide cofactor (Moco)^22^. Moco biosynthesis requires a variety of molybdoenzymes and molybdate^23^. In *Arthrobacter nicotinovorans*, MobA, which is responsible for the formation of Moco, was found to be essential for holoenzyme assembly of nicotine dehydrogenase and 6-hydroxypseudooxynicotine dehydrogenase^11^,^24^. In addition, SirA2, a sulfur-transferase in *Pseudomonas* sp. HZN6, was found to be associated with the hydroxylation of SP to HSP^25^.

Spm shows high similarity to other molybdenum-containing hydroxylases with respect to the amino acid composition of the three subunits. In our previous study, *spm* genes were expressed in *E. coli* leading to the formation of inactive Spm, whereas the organic configuration of the molybdenum cofactor in *E. coli* was found to be MGD^3^. The available form of Moco is Mo-MCD. Efforts to express and purify Spm in *Pseudomonas* have been met with little success; thus we could not directly test the content of Mo-MCD *in vivo*. To confirm that the deficiency of Mo-MCD caused the loss of Spm holoenzyme activity, we searched for genes related to Mo-MCD synthesis in *P. putida* S16. Here, we provide the first report of the gene *moaE*, which was found to be crucial for Mo-MCD synthesis and the nicotine metabolic pathway in *Pseudomonas*. The *moaE* gene deletion mutant had complete *spmABC* genes but could not convert SP to HSP, whereas the recombinant strain S16d*moaE*(pME6032-*moaE*) regained the ability to degrade SP. Site-directed mutagenesis suggested that the three conserved sites (Arg 37, Lys 117, Lys 124) were essential for enzyme activity of *moaE* which was consisted with previous report^16^. RT-qPCR was conducted to compare the transcriptional levels of *moaE, spmA*, and *spmC* in *P. putida* S16 grown in nicotine or glycerol medium. These three genes had relatively low mRNA levels, but appeared to be up-regulated in nicotine-induced *P. putida* S16. These results strongly suggest that *moaE* is induced in the presence of nicotine and plays an important role in SP conversion.

In summary, we cloned, sequenced, and characterized a novel gene in *Pseudomonas* that is crucial for the SP degradation involved in nicotine catabolism. The molybdenum cofactor biosynthetic gene was firstly reported in the pyrrolidine pathway of nicotine degradation. Thus these findings will expand our understanding of the molecular mechanisms of nicotine metabolism in *Pseudomonas*.

## Methods

### Chemicals and media

L-(-)-Nicotine (≥99% purity) was purchased from Fluka Chemie GmbH (Buchs, Switzerland). 3-Succinoylpyridine (SP) (98%) was obtained from Toronto Research Chemicals (Toronto, Canada). 6-Hydroxy-3-succinoylpyridine was prepared by our own laboratory^26^. All other chemicals were of analytical grade and commercially available. The “nicotine medium” was a minimal medium containing 13.3 g K_2_HPO_4_·3H_2_O, 4 g KH_2_PO_4_, 0.2 g MgSO_4_·7H_2_O and 0.5 ml of trace elements solution^27^. L-(-)-Nicotine was added to this minimal medium after filtration sterilization to a final concentration of 1 g l^−1^. The “glycerol medium” was a minimal medium added 1% glycerol and 1 g l^−1^ NH_4_Cl.

### Bacterial strains and culture conditions

*P. putida* S16 was isolated from tobacco cropping soil^28^ and cultured at 30 °C in the nicotine medium. *E. coli* cells and the gene deletion mutants of strain S16 were grown at 37 °C in Luria-Bertani (LB) medium, and kanamycin was used for selection at appropriate concentrations^27^.

### DNA manipulation and sequence analysis

Genomic DNA was isolated from strain *P. putida* S16 by using the Genomic DNA Purification Kit (LifeFeng, China). Purification of PCR products was performed with a Wizard Plus Minipreps DNA purification system (Promega, USA). Isolation of DNA fragments from agarose gels was accomplished with Gel Extraction Kit (Generay, China). Digestions with restriction endonucleases, ligations, and transformations were performed according to standard procedures. The sequences were analyzed with Vector NTI DNA analytical software (Invitrogen, USA) and homology searches were performed with the BLAST programs at the National Center for Biotechnology Information website (http://blast.ncbi.nlm.nih.gov/Blast.cgi). Phylogenetic tree of MoaE from several different strains was constructed using molecular evolutionary genetics analysis software MEGA 4.1 by neighbor joining (NJ) method and repeated bootstrapping for 1000 times was performed^15^.

### Construction of the *moaE* gene disrupted mutant *P. putida* S16d*moaE*

The *moaE* and other related genes in strain S16 were deleted by single homologous recombination (Fig. 2A). An internal fragment of *moaE* carrying 5′- and 3′ truncations was amplified by PCR and cloned into the multiply cloning site of pK18mob. PCR primer sequences were as follows: *moaEint*-EcoRI, CGGAATTCCGTTCGACCCGGGAGCCGAGAC and *moaEint*-HindIII, GCCAAGCTTTGGGTATTTTCCTTCTTCCAG. The recombinant plasmid pK18mob-*moaEint* was transferred into strain S16 by electroporation according to the following conditions: 0.5–1 μg DNA was added to 100 μl electrocompetent cells of *P. putida* S16, then the mixture was electroporated at 1.25 kVmm^−1^, 200 Ω, 25 μF with a Bio-Rad Gene-Pulser Xcell (Bio-Rad Laboratories, Hercules, CA). *P. putida* S16 exconjugants harboring disrupted genes were isolated on LB medium containing kanamycin and ampicillin. Other genes possibly related to molybdenum cofactor synthesis were disrupted as above. All the mutant strains were tested by PCR to confirm target gene disruption.

### Construction of complemented strain

Site-directed mutagenesis was performed by gene synthesis when three sites (Arg 37, Lys 117, Lys 124) were simultaneously replaced by Ala, the mutant of *moaE* gene was called *moaEmut*. The *moaE* gene and *moaEmut* gene were inserted into pME6032 and then transferred into strain *P. putida* S16d*moaE* by electroporation according to the above methods.

### Nicotine bioavailability assay

Strains *P. putida* S16, S16d*moaE*, S16d*moaE* (pME6032-*moaE*) and S16d*moaE* (pME6032-*moaEmut*) were incubated in 100 ml of LB medium containing 1 g l^−1^ of nicotine and 50 mg l^−1^ of ampicillin^4^. After 14 h vigorous shaking at 30 °C, cells were harvested by centrifugation (5,000 × g for 8 min at 4 °C) and washed twice with 50 mM phosphate-buffered saline (pH 7.0). The cells were then resuspended in the double-distilled water at OD_600 nm_ 5 (called resting cells). Nicotine was added to each batch of resting cells with the final concentration of 3 g l^−1^. The cell suspension was sampled during the reaction, the cells were removed by centrifugation at 12,000 × g for 1 min at 4 °C, and the supernatant was collected for analysis.

### General analytical techniques

The nicotine and 3-succinoylpyridine (SP) present in the culture medium were detected and quantified by high performed liquid chromatography (HPLC) (Agilent 1200 series) equipped with an Eclipse XDB-C18 column (column size, 250 mm × 4.6 mm; particle size, 5 μm; Agilent, USA). A mixture of methanol −1 mmol H_2_SO_4_ (10: 90 v v^−1^) was used as the mobile phase, at a flow rate of 0.5 ml min^−1^. Thin-layer chromatography (TLC) analysis was performed on 0.25 mm silica gel (GF254, JYD, Yantai, China) with a mobile phase consisting of chloroform: ethanol: methanol: H_2_O = 6: 3: 0.4: 0.3 (v v^−1^). The compounds on the gel were detected under an UV lamp (254 nm).

### RNA extraction and RT-qPCR

*P. putida* S16 was cultured in 5 ml nicotine medium from a single colony on nicotine medium plate. Afterwards, a 1: 50 dilution was inoculated with 50 ml nicotine medium (experimental group) and glycerol medium (control) in three 250 ml flasks. Cells were cultured at 30 °C with vigorous shaking and then harvested at the mid-exponential phase (OD_600 nm_ 0.9). Total RNAs were extracted from about 1 × 10^9^ cells of *P. putida* S16 by Total RNAprep cell/bacteria kit (Tiangen, China) and reverse transcribed to cDNA using random primers and SuperScript III reverse transcriptase (Invitrogen, USA)^3^. Quantitative PCR analysis was performed in the CFX96 Real-Time PCR Detection System (Bio-rad, USA) with SYBR GreenI RealMasterMix (Tiangen, China) and 10 fold diluted cDNA as the template. The qPCR primers were designed by Beacon designer and listed in Table 1. Melting curves and annealing temperature were confirmed by temperature gradient PCR. The standard curve for above-mentioned primers was performed with a tenfold dilution of *P. putida* S16 genomic DNA. The threshold cycle (C_T_) values for *spmA, spmC*, and *moaE* gene were measured referring to 16S rRNA gene, and the relative transcriptional level was reckoned by the 2^ΔΔCT^ method^29^.

### Nucleotide sequence accession number

The amino acid sequence reported in the present study has been deposited in the GenBank under the accession number AEJ14930.

## Additional Information

**How to cite this article**: Jiang, Y. *et al.* Functional Identification of a Novel Gene, *moaE,* for 3-Succinoylpyridine Degradation in *Pseudomonas putida* S16. *Sci. Rep.* **5**, 13464; doi: 10.1038/srep13464 (2015).

## Figures and Tables

**Figure 1:**
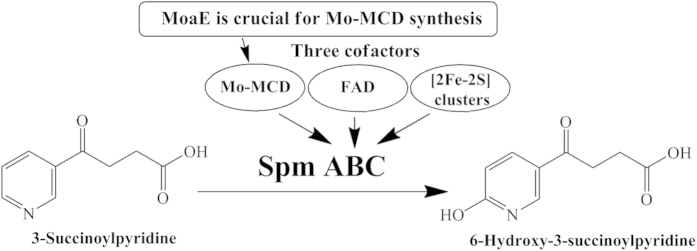
3-Succinoylpyridine degradation in *P. putida* S16. Spm catalyzes 3-succinoylpyridine (SP) to 6-hydroxy-3-succinoylpyridine (HSP) in the pyrrolidine pathway. Spm has three subunits SpmA, SpmB, SpmC, respectively binding three cofactors Mo-MCD, FAD, and [2Fe-2S] clusters. MoaE is crucial for Mo-MCD synthesis and Spm holoenzyme activity.

**Table 1:** Primers used in this study.

Primer	Sequence (5′-3′)
PPS_1556int-f	CGGAATTCCCAAGCTCGACCTTGACGATAT
PPS_1556int-r	GCCAAGCTTCGACGAACTGCTGCAGAAAGA
PPS_2936int-f	CGGAATTCCAGCAGGCCTACCAGGCGTATG
PPS_2936int-r	GCCAAGCTTCGCAGCAAGGCCTTCTGCAAA
PPS_4063int-f	CGGAATTCCAGTGGAGTACCTTAAGCAGG
PPS_4063int-r	CCCAAGCTTGTCAAGCAGAATTTGCTCGTA
*moaE*int-EcoRI	CGGAATTCCGTTCGACCCGGGAGCCGAGAC
*moaE*int-HindIII	GCCAAGCTTTGGGTATTTTCCTTCTTCCAG
Q-*spmA*-f	CCTATTCGCACTGGTATGG
Q-spmA-r	CTCACGCCTATCCTCAAC
Q-*spmC*-f	AGGAGCGGAGGTAGTTAG
Q-*spmC*-r	TGACAGTCCAGGTAATTCG
Q-*moaE*-f	CTTCATCATGGACTATCTG
Q-*moaE*-r	ATCACTCTGTTTCCCTTC
